# Chilblain-like lesion associated with coronavirus disease 2019 vaccine in tropical country: a case report

**DOI:** 10.1186/s13256-025-05254-7

**Published:** 2025-05-07

**Authors:** Wasuchon Chaichan, Mati Chuamanochan, Pongsak Mahanupab, Siri Chiewchanvit, Napatra Tovanabutra

**Affiliations:** 1 Division of Dermatology, Department of Internal Medicine, Faculty of Medicine, Chiang Mai University, 110 Intavarorot Road, Sriphum District, Maung, Chiang Mai, 50200 Thailand; 2 Department of Pathology, Faculty of Medicine, Chiang Mai University, 110 Inravarorot Road, Sriphum District, Maung, Chiang Mai, 50200 Thailand

**Keywords:** COVID-19 vaccine, Chilblain-like lesion, Chilblains, Lupus anticoagulant, Case report

## Abstract

**Background:**

Chilblains have emerged as a cutaneous manifestation following coronavirus disease 2019 vaccination. While there are many case reports on chilblain-like lesions, documentation from tropical countries remains limited. In this context, we report a case detailing chilblain-like lesions associated with coronavirus disease 2019 vaccination in Thailand.

**Case presentation:**

A 35-year-old Thai female patient presented with several painful, red papules on the fingers and toes 9 days after receiving the mRNA-1273 vaccination. Skin biopsy was performed, and the results were consistent with chilblains. Laboratory workup revealed positive result for lupus anticoagulant. The rash completely resolved without any treatment but reappeared following the second vaccine dose.

**Conclusion:**

Chilblain-like lesion following coronavirus disease 2019 vaccination is frequently observed in temperate countries, with few reports from tropical areas. The presence of lupus anticoagulant may contribute to the development of chilblains after coronavirus disease 2019 vaccination.

## Background

Chilblains are inflammatory cutaneous lesions characterized by painful, erythematous patches predominately affecting acral areas. They are commonly found in patients living in cold and damp environments [1]. During the coronavirus disease 2019 (COVID-19) pandemic, the incidence of chilblains increased in patients with COVID-19, and chilblains or chilblain-like lesions have been increasingly reported following COVID19 vaccination. The occurrence of chilblain in the context of COVID-19 or after COVID-19 vaccination may indicate a common mechanism involving the immune response targeting spike RNA or protein [2]. However, most reported cases were either from Europe or the USA. Here, we report a case of chilblain-like lesions associated with COVID-19 vaccination from Thailand.

## Case presentation

**Fig. 1:**
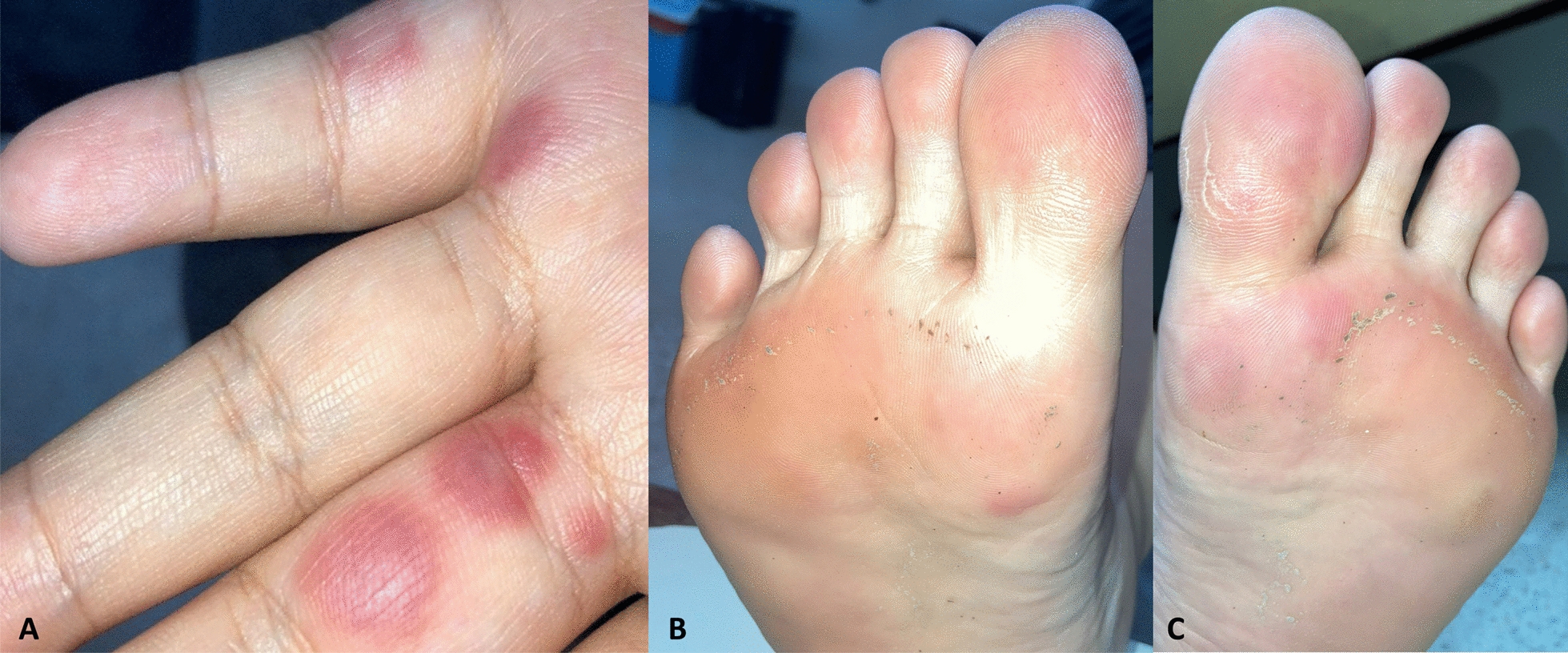
Multiple painful erythematous papules on the fingers (**A**) and toes (**B**, **C**)

A 35-year-old Thai woman presented to our institute on 4 December 2021 with multiple painful red bumps on her fingers and toes (Fig. 1), which appeared 9 days after her first dose of the mRNA-1273 vaccine (19 November 2021). She was previously healthy and had never been tested for COVID-19, nor did she have a history of COVID-19 infection. Additionally, she had never been exposed to cold and damp environments. She denied taking any medication or using substances. She had not sought any medical attention or received any treatment before this visit. There were no related medical conditions in her family. Physical examination showed multiple discrete erythematous papules on fingers, both feet, and toes. No other remarkable findings were noted. The presumptive diagnosis was Sweet syndrome. A punch biopsy of right palm was obtained, and the patient was prescribed oral colchicine 0.6 mg twice daily.

**Fig. 2:**
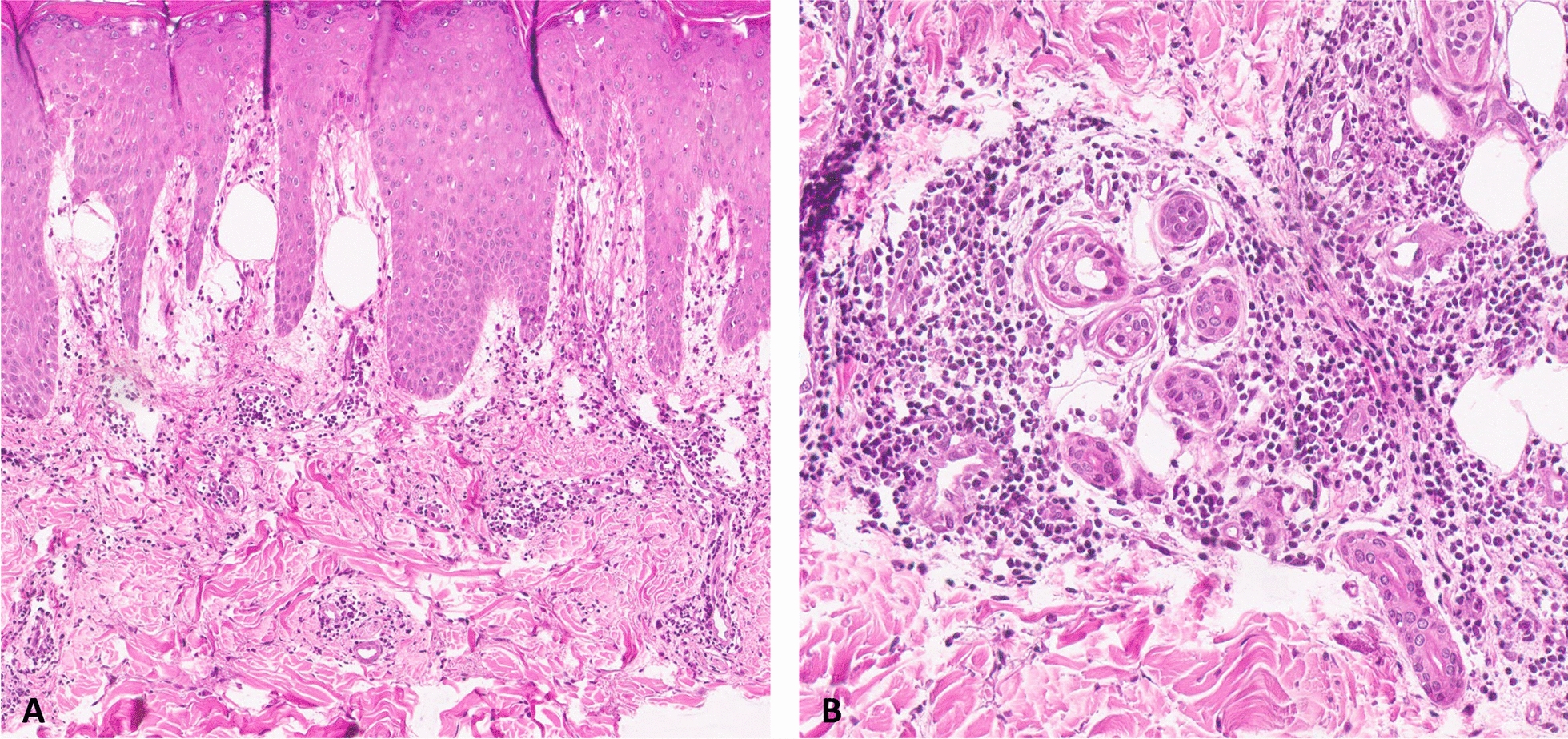
Histologic findings of the skin punch biopsy showing papillary dermal edema, perivascular mononuclear cell infiltration (**A**), and perieccrine lymphocytic infiltration (**B**)

Laboratory workups showed normal results for complete blood count, antinuclear antibody, erythrocyte sedimentation rate, C-reactive protein, anti-cardiolipin, and anti-β2 glycoproteins, with the exception of a positive result for lupus anticoagulant. Histopathology revealed papillary dermal edema, superficial and deep perivascular mononuclear cell infiltration (Fig. 2A), and perieccrine lymphocytic infiltration (Fig. 2B), which were consistent with chilblain.

Complete resolution of the rash was observed during the 2-week follow-up visit (16 December 2021). However, following the administration of her second vaccine dose on 18 December 2024, a similar rash appeared at the same site. Regarding the laboratory test, the lupus anticoagulant remained positive at the 3-month follow-up visit.

## Discussion

Chilblains are uncommon in tropical countries and can be categorized as primary and secondary. Primary chilblains are associated with abnormal responses to cold, resulting in vasoconstriction in acral sites [1]. Secondary chilblains are linked to autoimmune connective tissue diseases, cryoglobulinemia, hyperviscosity syndrome, antiphospholipid syndrome, and leukemia [3]. Chilblains are a common cutaneous manifestation of COVID-19, and a few cases of chilblains have also been reported following mRNA COVID-19 vaccination.

The mechanism of chilblains in COVID-19 involves the immune response targeting viral spike RNA or protein, leading to the activation of type I interferon [4]. This activation can interfere with immune tolerance, provoke an autoimmune response, and inhibit the endothelial nitric oxide synthase pathway, which could be a possible cause of vasospasm in chilblains [5]. The immune response was triggered by the COVID-19 mRNA vaccine, possibly similar to the response after COVID-19 infection, which can also triggers chilblains [2]. Histopathology of chilblains following COVID-19 mRNA vaccination demonstrated high level of myxovirus resistance protein A in the skin lesion. This suggests a locally increased activation of interferon type I pathway, as observed in COVID-19 associated chilblains [6]. Although our patient did not undergo testing for COVID-19 infection before the appearance of chilblains, she denied any symptoms consistent with COVID-19 infection. In this case, the chilblains were triggered by COVID-19 vaccination, as confirmed by their recurrence after the second dose of the vaccine.

The association between chilblains and antiphospholipid antibodies has been observed in several case reports, involving mainly young women, and chilblains can be either an initial sign or present after the diagnosis of antiphospholipid syndrome [7–9]. A retrospective study revealed a higher rate of chilblains in the patients with antiphospholipid syndrome, with hazard ratio of 1.82 [10]. Additionally, several reports have documented positive antiphospholipid antibodies in approximately half of patients with COVID-19 infections, and these antibodies can be detected after COVID-19 vaccinations [11–13]. However, their relationship remains unclear [14, 15].

The production of antiphospholipid antibodies could potentially be explained by a mechanism involving molecular mimicry. Furthermore, mRNA vaccines may activate the coagulation cascade and interfere with the interaction between platelets and endothelial cells [16]. This interaction disturbance may trigger chilblains in a patient who either has preexisting antiphospholipid antibodies or develops *de novo* antibody positivity.

Before the COVID-19 pandemic, our patient did not undergo testing for antiphospholipid antibodies due to the absence of symptoms and signs of antiphospholipid syndrome. Therefore, it is difficult to determine whether this case involved preexisting antiphospholipid antibodies or *de novo* development following COVID-19 vaccination. However, as antiphospholipid antibodies are present in 1–5% of the normal population, it is possible that our patient had preexisting lupus anticoagulant, and COVID-19 vaccination provoked the development of chilblains in the context of a tropical climate [17].

Currently, treatment recommendation for chilblain-like lesions associated with COVID-19 vaccination is not available. However, the case series has revealed that management of this patient group varied, ranging from no treatment to smoking cessation, nonsteroidal antiinflammatory drug, and glucocorticoid [14]. In our patient, the lesions showed spontaneous remission.

## Conclusion

Here we report a case of chilblain-like lesion due to mRNA-1273 vaccine. Although they are frequently observed in temperate counties, the reports from tropical areas are limited. The presence of lupus anticoagulant may contribute to the development of chilblains after COVID-19 vaccination. Given the limitation of this finding to a single case, additional case reports, or ideally, prospective studies in similar contexts are needed to better understand the role of antiphospholipid antibodies in patients who develop chilblains following COVID-19 vaccination.

## Data Availability

The datasets used and/or analyzed during the current study are available from the corresponding author on reasonable request.
